# The Spread of Sleep Loss Influences Drug Use in Adolescent Social Networks

**DOI:** 10.1371/journal.pone.0009775

**Published:** 2010-03-19

**Authors:** Sara C. Mednick, Nicholas A. Christakis, James H. Fowler

**Affiliations:** 1 Department of Psychiatry, University of California San Diego, La Jolla, California, United States of America; 2 Department of Medicine, Harvard University, Boston, Massachusetts, United States of America; 3 Department of Political Science, University of California San Diego, La Jolla, California, United States of America; Chiba University Center for Forensic Mental Health, Japan

## Abstract

Troubled sleep is a commonly cited consequence of adolescent drug use, but it has rarely been studied as a cause. Nor have there been any studies of the extent to which sleep behavior can spread in social networks from person to person to person. Here we map the social networks of 8,349 adolescents in order to study how sleep behavior spreads, how drug use behavior spreads, and how a friend's sleep behavior influences one's own drug use. We find clusters of poor sleep behavior and drug use that extend up to four degrees of separation (to one's friends' friends' friends' friends) in the social network. Prospective regression models show that being central in the network negatively influences future sleep outcomes, but not vice versa. Moreover, if a friend sleeps ≤7 hours, it increases the likelihood a person sleeps ≤7 hours by 11%. If a friend uses marijuana, it increases the likelihood of marijuana use by 110%. Finally, the likelihood that an individual uses drugs increases by 19% when a friend sleeps ≤7 hours, and a mediation analysis shows that 20% of this effect results from the spread of sleep behavior from one person to another. This is the first study to suggest that the spread of one behavior in social networks influences the spread of another. The results indicate that interventions should focus on healthy sleep to prevent drug use and targeting specific individuals may improve outcomes across the entire social network.

## Introduction

In 2006, 15.7% of 8th-graders and 42.3% of 12th-graders had tried marijuana at least once, and about 18% of 12th-graders were current users in the United States [1]. Inability to sleep and excessive sleepiness are often cited as primary warning signs and symptoms of such teenage drug abuse [2]. The implication is that drug abuse causes sleep problems. Indeed, in previous studies of adolescent use of opioids [3], alcohol [4], and marijuana [5], [6], [7], [8], researchers have generally assumed that the causal direction is from substance abuse to sleep problems.

Yet, one of the biggest adjustments affecting late adolescence is the significant change in chronotype with the delay of the intrinsic sleep phase [9]. Coping with delayed sleep phase becomes problematic for teens who need to wake up early for morning classes, resulting in average weeknight sleep durations around 7 hours per night [10]–much less than the 8.50–9.25 hours needed at this phase of their lives [11].

Such poor sleep might lead to drug use. According to one of the few longitudinal studies, young teenagers with poor preschool sleep habits were more than twice as likely to use drugs, tobacco, or alcohol ten years later, even after controlling for issues such as depression, aggression, attention problems and parental alcoholism [12]. A more recent experimental study had teen subjects sleep for two weeks in a long sleep condition (10 hr/night) and two weeks in a short-sleep condition (6.5 hr/night). Parents and teens both reported that participants in the short sleep condition had many more behavioral, cognitive, and emotional problems [13]. In turn, conduct problems are cited as one of a number of factors that precede and correlate with substance use in teens [14].

Also relevant to adolescent drug use, however, are social factors such as family and school functioning [15] and peer pressure [16]. Adolescents are embedded in complex social networks and are especially vulnerable to peer effects, possibly not only with respect to drugs but also with respect to sleep. An unexplored aspect of the connection between adolescent sleep and drug abuse is therefore the impact of social networks. Many behaviors and affective states, including smoking [17], drinking [18], weight gain [19], loneliness [20], depression [21], and happiness [22], have recently been shown to spread through social networks among adults followed for many years. It seems likely that both sleep habits and drug use could also spread among adolescents. If so, the two behaviors, as evinced among members of peer groups, might in turn influence each other–between individuals and not just within them.

Here, we use nationally representative data to show that both sleep behavior and marijuana use spread through social networks and mediation analyses suggest that an adolescent's sleep behavior influences a friend's marijuana usage via the impact on the friend's sleep behavior. This set of relationships suggests that the causal arrow points from sleep to drug use rather than the other way around.

## Materials and Methods

### The Add Health Data

The National Longitudinal Study of Adolescent Health (*Add Health*) is a nationally-representative sample of students in grades 7–12.[23] At the beginning of Wave I, researchers identified an “in-school” sample of 90,118 adolescents in 142 schools. These students filled out questionnaires and named up to 5 male and 5 female friends who were later identified from school-wide rosters to generate information about each school's complete social network. A subset of this group was then chosen for in-depth follow-up later in Wave I (1994–1995), and also in Waves II (1996), and III (2001–2002). This “in-home” sample was administered a longer questionnaire about their networks and health behavior from which we draw our information about sleep and drug use. The average age at inception of the group was 15.8 (SD 1.6), and 51% were female; students averaged 7.8 hours of sleep per night (SD 1.4) and used marijuana an average of 1 time (SD 4.5) in the past month (see Text S1). We analyzed only Wave I and II data here, as, by Wave III, the subjects were no longer in school.

We treat each friendship nomination as a “directed tie” from the namer to the named friend. We call individuals who are the objects of analysis “egos” and the people to whom they are connected “alters.” Sleep was assessed by self-report at both waves (“How many hours of sleep do you usually get?”). Although the validity of self-report of sleep habits is controversial [24], one study of adolescents comparing self-report to actigraphy reported good correlation [25]. For most analyses, we dichotomize the sleep variable by dividing the sample into those who get more than 7 hours (62% of the sample at Wave I and 56% at Wave II) and those who get 7 or less (38% and 44%, respectively).

Marijuana use was also assessed by self-report at both waves (“During the past 30 days, how many times did you use marijuana?”). Studies comparing self-report to biochemical assessments have shown high accuracy of self-report in tobacco-smoking adults [26] and marijuana-using adolescents [27]. For most analyses, we dichotomize the marijuana variable by dividing the sample into those who used at least once (13% of the sample at Wave I and 15% at Wave II) and those who did not use at all (87% and 85%, respectively). For analyses of the number of times used, we truncate the maximum value to 30 (representing average usage of once a day).

### Statistical Analyses

An association in the behaviors of connected individuals can be attributed to at least three processes: 1) *influence*, whereby a behavior in one person causes the behavior of others; 2) *homophily*, whereby individuals with the same behaviors preferentially choose one another as friends [23]; or 3) *confounding*, whereby connected individuals jointly experience contemporaneous exposures (such as a noisy neighbourhood or a local drug dealer). To distinguish among these effects requires repeated measures of sleep and drug use [27], longitudinal information about network ties, and information about the nature or direction of the ties (e.g., who nominated whom as a friend).

For some analyses, we considered the prospective effect of social network variables (such as network centrality–which measures how central a person is in a network (see Text S1)) and friends' overall behavior on ego's future sleep behavior and drug use. For other analyses, we conducted regressions of ego sleep behavior or drug use in Wave II as a function of ego's age, gender, race, ethnicity, household income, parental education, and sleep or drug behavior in Wave I, and of the sleep or drug behavior of an *alter* in both Wave II and Wave I. Inclusion of ego's behavior at Wave I helps to control for ego's genetic endowment and any intrinsic, stable predilection to engage in drug use or to sleep. Including alter's behavior at Wave I helps control for homophily [23]. The key coefficient in these models that measures the effect of influence is the variable for alter's Wave II behavior [23].

We estimated both logit models (where we consider a dichotomous version of the outcome variable) and normal specifications (where we consider a continuous version of the variable [hours of sleep, number of uses of marijuana]). We use generalized estimating equation (GEE) procedures to account for multiple observations of the same ego across ego-alter pairings [28] and we assume an independent working correlation structure for the clusters [28]. Huber-White sandwich estimates with clustering on the egos yielded very similar results.

The GEE regression models in the tables provide parameter estimates in the form of beta coefficients, whereas the results reported in the text and figures are in the form of risk ratios. Mean effect sizes and 95% confidence intervals were calculated by simulating the first difference in alter's Wave II behavior (changing from getting >7 hours of sleep to ≤7 hours of sleep, or from no drug use to some drug use) using 1,000 randomly drawn sets of estimates from the coefficient covariance matrix and assuming all other variables are held at their means [28].

We evaluated the possibility of omitted variables or contemporaneous events explaining the associations by examining how the type or direction of the social relationship between ego and alter affects the association. If unobserved factors drive the association between ego and alter, then direction of friendship should not be relevant. We also considered the possibility that school contextual effects may drive our results, and analyses (available in Text S1) show that our results remain significant and effect sizes are similar in models that restrict observations to a single school. Finally, we performed a mediation analysis in which we test the hypothesis that alter's sleep influences ego's sleep which in turn has an effect on ego's drug use (see Text S1 for more details) [29], [30].

## Results

### Sleep and Drug Behavior Associations Extend Up to Four Degrees of Separation

**Figure 1:**
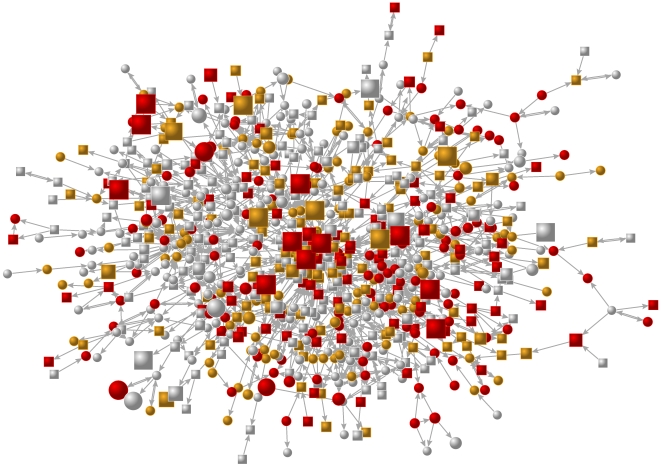
Network graph. Figure 1 is a network graph of the largest component of friends in Wave I of the Add Health study (year 1995), from a single school. Each node represents a subject (there are 800 shown) and its shape denotes gender (circles are female, squares are male). Lines between nodes indicate relationships (arrows point from the naming friend to the named friend). Node colour denotes nightly sleep duration (red for 6 hours or less, orange for 7 hours, white for 8 hours or more) and node size indicates frequency of marijuana use (the smallest nodes do not use marijuana, the largest report using at least daily). The network suggests clustering of both sleep and drug use behavior, and as we show in the statistical analysis, some of the overlap in clustering may result from a causal effect of sleep on drug use. Node placement is based on the Kamada-Kawai algorithm (see Text S1) [50].

**Figure 2:**
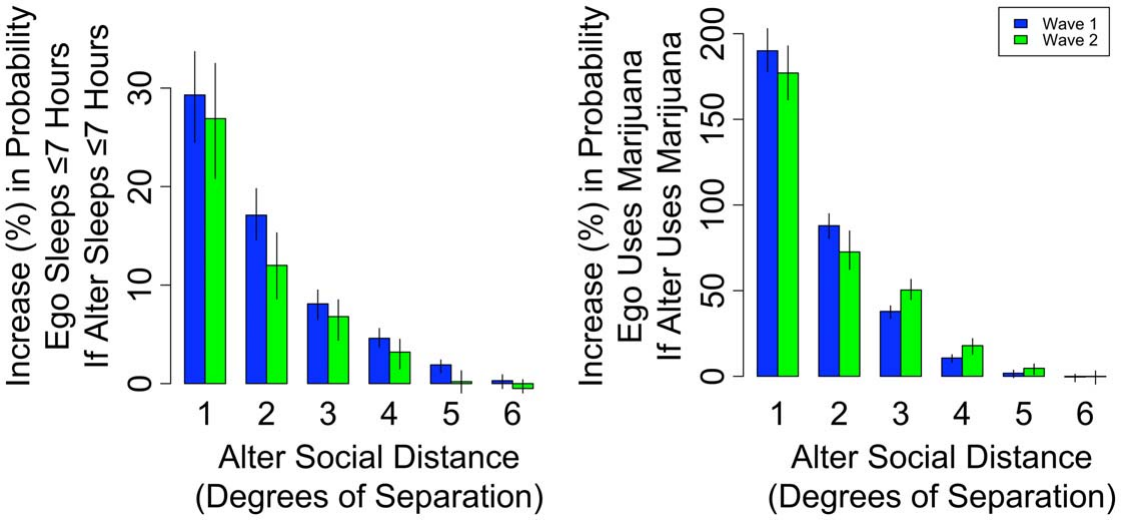
Spread of sleep and drug use. Figure 2 shows that the association between ego and alter sleep is significant up to four degrees of separation in both Wave I and Wave II. The left panel shows the percentage increase in the likelihood a person sleeps ≤7 hours if a friend at a certain social distance sleeps ≤7 hours. The right panel shows the percentage increase in the likelihood a person uses marijuana if a friend at a certain social distance uses marijuana. The relationship is strongest between individuals who are directly connected, but it remains significantly greater than zero at social distances up to 4 degrees of separation in both Wave I and Wave II. Thus, a person's sleep and drug use behavior is associated with the same behavior of other people up to 4 degrees removed from them in the network. Error bars are derived by comparing the conditional probability of the behavior in the observed network with an identical network in which topology and incidence of the behavior is preserved but the assignment of the behavior is randomly distributed [19,55 2008]. Alter social distance (degrees of separation) refers to closest social distance between the alter and ego (friend  =  distance 1, friend's friend  =  distance 2, *etc.*). Error bars show 95% confidence intervals.

Figure 1 shows the largest connected network component in the largest school in Wave I, based on ties among friends (sibling ties are excluded to simplify the image). The clusters of poor sleep (≤7 hrs) subjects seen in the network are significantly larger than expected due to chance. Figure 2 shows that the association between ego and alter sleep is significant up to four degrees of separation in both Wave I and Wave II. For example, in Wave I a subject is 29% (95% C.I. 24% to 34%) more likely to sleep ≤7 hours if a directly connected alter (distance 1) sleeps ≤7 hours. The relationship for distance 2 alters (the friend of a friend, i.e. two degrees of separation) is 17% (14% to 20%), for distance 3 alters is 8% (6% to 10%), and for distance 4 alters is 5% (4% to 6%). Figure 2 also shows the results using the same procedure for drug use. In Wave I a subject is 190% (178% to 203%) more likely to use marijuana if a directly connected alter (distance 1) uses marijuana. The relationship for distance 2 alters is 88% (80% to 95%), for distance 3 alters is 38% (34% to 41%), and for distance 4 alters is 11% (8% to 13%).

### Network Centrality and Future Sleep and Drug Use

Figure 1 also suggests a relationship between network centrality, sleep, and drug use: subjects at the core of the networks appear to sleep less and to be more likely to use marijuana. We tested these relationships more rigorously by computing network centrality for each subject (see Text S1). We found that centrality is significantly associated with less sleep in the future: a two-standard-deviation increase in centrality at Wave I increases the probability of sleeping ≤7 hours at Wave II by 13% (95% CI: 1% to 26%, *p* = 0.03). This relationship between centrality and sleep is net of controls for age, race, ethnicity, household income, and mother's education.

In contrast, we find no significant relationship between centrality and drug use (see Text S1). We also considered the possibility of effects in the reverse direction, examining the impact of sleep and drug use in Wave I on network centrality in Wave II. We find that neither sleep nor marijuana use has an effect on future network centrality. Thus, it appears that, in the case of sleep, it is the network structure that influences the behavioral outcome and not vice versa.

### Spread of a Single Behavior Across Time: Relationship Between Alter Sleep and Drug Use and Ego Behaviors

Ego sleep behavior in Wave II is associated with alter sleep behavior in Wave I, as shown in Table 1. Notably, each additional contact who sleeps ≤7 hours in Wave I increases the likelihood the ego will sleep ≤7 hours in Wave II by 5% (95% CI: 1% to 10%, *p* = 0.02). In a continuous model with hours of sleep as the dependent variable, the effect is also significant (*p* = 0.03). In contrast to the spread of poor sleep behavior, the number of contacts who sleep >7 hours has a weak but not-quite significant (*p* = 0.08) positive effect on an individual's sleep behavior.

**Table 1:** Sleep and social contacts.

*Dependent Variable: Respondent Wave II Sleep Behaviour*	*Sleep ≤7 Hours*			*Hours of Sleep*		
	Coef	SE	p	Coef	SE	p
*No. Contacts Who Sleep >7 Hours at Wave I*	−0.03	0.02	0.08	0.02	0.01	0.06
*No. Contacts Who Sleep ≤7 Hours at Wave I*	0.05	0.02	0.02	−0.03	0.01	0.03
*Age*	0.20	0.02	0.00	−0.12	0.01	0.00
*Female*	0.11	0.05	0.02	−0.11	0.03	0.00
*Household Income*	0.00	0.00	0.58	0.00	0.00	0.72
*Mother's Education*	0.03	0.01	0.00	−0.02	0.01	0.00
*Hispanic*	0.04	0.07	0.54	−0.05	0.01	0.20
*Black*	0.17	0.06	0.01	−0.12	0.04	0.00
*Asian*	0.07	0.10	0.50	−0.02	0.04	0.76
*Respondent Sleep Behaviour at Wave I*	1.30	0.05	0.00	1.30	0.05	0.00
*Constant*	−4.35	0.29	0.00	3.00	0.30	0.00
*Deviance*	10294			10294		
*Null Deviance*	11470			11470		
*N*	8349			8349		

Table 1 shows spread of sleep behavior. Results for logistic regression of ego sleep behavior at Wave II (1 =  sleeps ≤7 hours, 0 =  sleeps >7 hours) on Wave I covariates is shown in first three columns. Results for ordinary least squares regression of ego sleep behavior at Wave II (dependent variable is total hours slept) on Wave I covariates is shown in second three columns. The results suggest that poor sleep behaviors may be more likely to spread than good sleep behaviors.

Similarly, ego drug use in Wave II is associated with the number of contacts in Wave I who use drugs, as shown in Table 2. Each additional drug-using friend increases the likelihood of use by 42% (95% CI: 28% to 57%) at the next wave. And each additional drug-using friend increases the number of uses in the previous 30 days by 0.45 uses (0.31 to 0.59), which may be compared with the average number of uses of 1.06. Non-drug-using friends also have a negative effect on use, but the effect is smaller. Each non-using friend decreases the likelihood of use by 10% (4% to 15%) and also decreases the number of uses in the past 30 days by 0.11 (0.05 to 0.17). In both models, the magnitude of the coefficient on the number of non-users is significantly smaller than the magnitude of the coefficient on the number of users (*p*<0.001). Thus, like sleep behavior, the spread of drug-using behavior exhibits an asymmetry that suggests the negative health behavior is more contagious than the positive health behavior.

**Table 2:** Marijuana use and social contacts.

*Dependent Variable: Respondent Wave II Marijuana Behaviour*	*Recently Used*			*Number of Uses*		
	Coef	SE	p	Coef	SE	p
*No. Contacts Who Used Marijuana at Wave I*	0.35	0.05	0.00	0.45	0.07	0.00
*No. Contacts Who Did Not Use at Wave I*	−0.10	0.03	0.00	−0.11	0.03	0.00
*Age*	0.13	0.04	0.00	0.10	0.03	0.00
*Female*	−0.48	0.11	0.00	−0.46	0.10	0.00
*Household Income*	0.00	0.00	0.14	0.00	0.00	0.02
*Mother's Education*	0.00	0.03	0.94	0.04	0.02	0.11
*Hispanic*	−0.06	0.16	0.68	0.06	0.14	0.68
*Black*	−0.81	0.18	0.00	−0.56	0.13	0.00
*Asian*	−0.41	0.27	0.12	−0.19	0.20	0.35
*Respondent Marijuana Behaviour at Wave I*	3.13	0.14	0.00	0.56	0.01	0.00
*Constant*	−4.47	0.67	0.00	−0.04	0.55	0.95
*Deviance*	2707			150928		
*Null Deviance*	3411			207660		
*N*	8128			8128		

Results for logistic regression of ego drug use behavior at Wave II (1 =  used marijuana in past 30 days, 0 =  did not use) on Wave I covariates is shown in first three columns. Results for ordinary least squares regression of number of times ego uses marijuana at Wave II on Wave I covariates is shown in second three columns. The results suggest that both drug use and non-use may spread, but the spread of use is significantly stronger.

**Figure 3:**
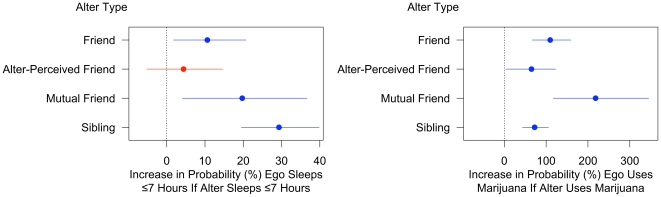
Influence of friends and siblings. Figure 3 shows that friends and siblings significantly influence drug use and sleep behavior. Effects are estimated using generalized estimating equation (GEE) logit models of sleep and drug use on several different sub-samples of the Add Health social network; see Text S1 for the underlying models.

We next examined how the actual behavior in ego's alters was associated with ego's sleep behavior and drug use. Unlike the prospective models which evaluated the impact of the overall count or number of a person's friends who behaved a particular way, these models look at ego-alter pairs and examine how a change in behavior in each alter is associated with a change in behavior in the ego. The models adjust for sex, age, race, ethnicity, household income, parental education, and the ego's prior behavior. Including alter's prior behavior helps control for the process of selecting friends based on their behaviors. However, our main interest here is the impact of one person's behavior on the behavior of others, which we estimate using the coefficient on alter's current behavior. Figure 3 shows the results of these models, and how they vary for different kinds of friends and for siblings (see Text S1).

If a friend sleeps ≤7 hours, it increases the likelihood the ego sleeps ≤7 hours by 11% (95% CI: 2% to 21%). Similarly, if a friend becomes a marijuana user, it more than doubles the likelihood the ego uses marijuana (110% increase, 67% to 159%). When we analyze how often subjects used marijuana in the past 30 days as the dependent variable, we find that each use by a friend is associated with 0.14 additional uses by the ego (0.10 to 0.18). To be sure that between-school differences were not responsible for the effect, we restricted the sample to a single, large school, with the same results (see Text S1).

One concern in network studies is the possibility that contextual effects can confound the analysis. The finding that the results are robust within a single school helps to mitigate this concern, but an additional technique is to take advantage of the *directionality* of friendships. Each person was asked to name their friends, but not all of these nominations were reciprocated. Therefore, we can distinguish three possibilities: ego-perceived friends (denoted “friends”), “alter-perceived friends” (the alter named ego as a friend, but not vice versa), and “mutual friends” (ego and alter nominated each other). If the association between ego and alter is driven by a shared exposure (such as a local drug dealer), we would expect the effects to be equally strong in each of these types of relations. But if the association is due to influence, then we would expect ego-perceived friends to be more influential then alter-perceived friends, since, in the latter case, the ego may not even know a person who has named them as a friend. Moreover, we expect mutual friends to be the most influential.

In Figure 3, we show that the effect of alter-perceived friends on sleep is not significant (*p* = 0.35) and on drug use is marginally significant (65%, 95% CI: 4% to 123%) but about half the size of the effect of ego-perceived friends. Moreover, mutual friends exhibit the strongest associations. A mutual friend who sleeps ≤7 hours increases the likelihood the ego sleeps ≤7 hours by 20% (4% to 37%) and a mutual friend who starts using marijuana more than triples the risk that the ego uses marijuana (218% increase,117% to 345%). These results suggest that at least part of the association between ego and alter behavior is due to influence.

Finally, a sibling who sleeps ≤7 hours increases the likelihood that the ego sleeps ≤7 hours by 29% (20% to 40%) and a sibling who starts using marijuana increases the likelihood the ego uses by 73% (44% to 105%). However, it is important to note that unlike friends, sibling relationships are all mutual, so we cannot use directionality to test whether these associations result from influence, shared genes, or a shared home environment. We present results for siblings primarily for comparison to the friend effects.

### Mediation Effects Between Sleep and Drug Use

In Table 3, we present the results of a mediation analysis in which the hypothesized causal pathway is that a friend's sleep behavior can spread to the ego, and the resulting *change* in ego's sleep behavior can also affect ego's drug-using behavior, net of any predilection to form friendships based on sleep, drug use, or other factors. The results show that when a friend sleeps ≤7 hours it increases the likelihood that the ego will use marijuana by 19% (95% CI: 2% to 39%, *p* = 0.02). Moreover, in keeping with the baseline analyses described above, alter's sleep behavior is significantly associated with ego's sleep behavior (*p* = 0.001), and ego's marijuana use is significantly associated with ego's sleep behavior, controlling for alter's sleep behavior (*p* = 0.001). When we estimate the size of the mediated effect, we find that when a friend sleeps ≤7 hours, it increases the likelihood that the ego will use marijuana by 4% (1% to 7%) via its impact on ego's sleep behavior. This represents approximately 20% of the total effect of alter sleep on ego drug use. It is important to note that this effect is additive; each *additional* friend who sleeps poorly also significantly increases drug use by the same amount.

**Table 3:** Ego sleep mediates relationship between alter sleep and ego drug use.

*Dependent Variable:*	*Ego Currently Uses Marijuana*			*Ego Currently Sleeps ≤7 Hours*			*Ego Currently Uses Marijuana*		
	Coef	SE	p	Coef	SE	p	Coef	SE	p
*Alter Currently Sleeps ≤7 Hours*	0.20	0.09	0.02	0.19	0.06	0.00	0.19	0.09	0.03
*Ego Currently Sleeps ≤7 Hours*	-----	-----	-----	-----	-----	-----	0.35	0.11	0.00
*Alter Previously Slept ≤7 Hours*	0.08	0.09	0.35	0.14	0.07	0.04	0.07	0.09	0.42
*Ego Previously Slept ≤7 Hours*	0.35	0.10	0.00	1.48	0.08	0.00	0.23	0.11	0.04
*Ego Female*	0.21	0.10	0.03	0.15	0.08	0.05	0.22	0.10	0.03
*Ego Age*	0.14	0.03	0.00	0.20	0.03	0.00	0.13	0.03	0.00
*Ego's Household Income*	−0.00	0.00	0.05	0.00	0.00	0.49	−0.00	0.00	0.05
*Mother's Education*	0.01	0.02	0.57	−0.01	0.02	0.62	0.01	0.02	0.55
*Ego Hispanic*	−0.03	0.15	0.83	−0.22	0.12	0.06	−0.01	0.15	0.91
*Ego Black*	−0.35	0.14	0.02	0.11	0.10	0.27	−0.36	0.14	0.01
*Ego Asian*	−0.54	0.21	0.01	0.22	0.14	0.13	−0.55	0.21	0.01
*Constant*	−4.02	0.58	0.00	−4.45	0.49	0.00	−3.89	0.59	0.00
*Deviance*	689			1224			687		
*Null Deviance*	702			1465			702		
*N*	5913			5913			5913		

Table 3 shows mediation effect of sleep on drug use. First three columns show logistic regression model of ego's marijuana use behavior (the outcome variable) on alter's sleep behavior (the explanatory variable). Second three columns show logistic regression model of ego's sleep behavior (the mediator variable) on alter's sleep behavior (the explanatory variable). Last three columns show logistic regression model of ego's marijuana use behavior (the outcome variable) on ego's sleep behavior (the mediator variable) controlling for alter's sleep behavior (the explanatory variable). Models were estimated using a general estimating equation with clustering on the ego and an independent working covariance structure. Models with an exchangeable correlation structure yielded poorer fit. Fit statistics show sum of squared deviance between predicted and observed values for the model and a null model with no covariates. A bootstrap procedure that takes into account uncertainty of both the effect of the independent variable on the mediator and the mediator on the outcome variable shows that when an alter starts sleeping ≤7 hours, it increases the likelihood of ego drug use by 4% (95% C.I. 1% to 7%) via its effect on the mediator (ego sleep), which represents approximately 20% of the total effect of alter sleep on ego drug use.

Finally, we also tested the hypothesis that the relationship between alter sleep and ego drug use is mediated by the spread of drug use, rather than sleep. While the coefficients are all at least marginally significant in the individual models (see Text S1), the mediated effect is not. We also reversed the direction of the hypothesis and studied whether alter's drug use influences ego's sleep behavior, via either the spread of drug use or the spread of sleep behavior. In neither case did we find a significant direct association (see Text S1), suggesting the causal pathway is from sleep to drugs and not the other way around.

## Discussion

**Figure 4:**
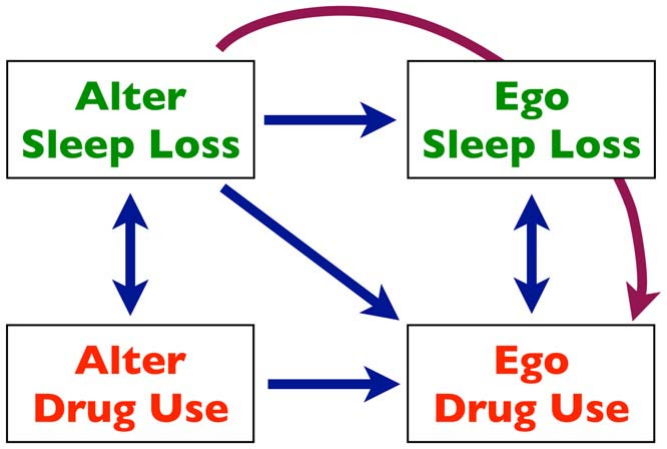
Summary of results. Blue arrows indicate significant direct relationships, while the purple arrow indicates the only mediation relationship that was found to be significant between alter sleep behavior and ego drug use. Arrows from alter to ego are directional because the statistical evidence suggests that at least some of the associations result from a causal relationship.

Figure 4 summarizes the main findings. The evidence suggests that poor sleep leads to drug use in adolescents, and that both sleep and drug use spread through social networks. We show (for the first time, to our knowledge) a mediation effect in which the spread of one health behavior affects the spread of another. Specifically, a person's risk of drug use increases if his friends sleep poorly, and this effect is mediated in part by the spread of poor sleep behavior from one person to another.

It is well known that both sleep and drug use can be influenced by social pressure [31], [32]. But behaviors do not exist in isolation: one behavior can influence another both within and between individuals. Hence, beyond social influence, the present study is the first to consider the coevolution of two spreading processes on a fully observed network. By tracking subjects over time, our data show not only the pattern of growth of two independent health behaviors, but go further to reveal a mechanism by which one may encourage another. One possible explanation for our results may be the effect of poor sleep on decision-making and behavior regulation. Poor sleep in adolescents has been shown to decrease behavior regulation, including impulse control, emotion regulation, and behavioral flexibility [13], as well as health-risk behaviours, such as suicidality and substance use [33], and poor school performance [9], [34].

The present study was limited to survey questions gathered about marijuana use. It is common, however, for marijuana to act as a ‘gateway’ increasing risk for youth to progress to other, more serious drugs [35], [36]. The National Center on Addiction and Substance Abuse at Columbia University recently reported that nearly half of full-time students binge drink and/or abuse prescription and illegal drugs [37]. From 1993 to 2005, abuse of prescription opioids, or pain killers, increased 343%; abuse of prescription tranquilizers such as Xanax and Valium rose 450%; and abuse of prescription stimulants such as Adderall was up 93.3%. The present study was unable to account for the effect of other substance use on an individual's sleep patterns, or the progression of friends' substance use on the network. Examination of the spread of other substances, such as amphetamines that are known to disrupt sleep, would be a natural extension of the present findings.

We also show a number of novel network effects. In contrast to prior studies that showed associations in behaviors and affective states extending up to three degrees of separation [17], [18], [19], [20], [21], [22], [38], here we show they extend up to four degrees for both sleep and drug use. We also show that people with high network centrality are at greater risk of poor sleep, which in turn affects drug use. This is consistent with the finding that negative outcomes for both sleep and drug use spread more reliably than positive ones; more centrally located individuals are in a structural location that makes them more susceptible to behavioral contagions, since they sit at a sort of “crossroads” in the network.

These results pave the way for further analysis of other negative health outcomes that may be mediated by sleep, such as alcohol use. It is likely that the negative effects of poor sleep that lead an adolescent to try marijuana would influence decisions to try alcohol in similar ways. We also hypothesize that behaviors that have already been shown to spread through social networks, such as obesity [19], drinking [18], and smoking [17], may be good candidates for such a mediation analysis. And, apart from sleep, these data suggest a way forward in identifying the causal processes that underlie important behavioral contagions. For example, we could use the same methodology to study whether the spread of eating certain types of food (like junk food) mediates the spread of obesity [19].

Our findings suggest a number of directions that might help policy-makers and researchers to improve anti-drug campaigns. First, efforts to increase sleep in adolescents may help to reduce substance use in this population. Although the present findings show that poor sleep behavior has a stronger influence on the network compared to good sleep behavior, lessening the influence of poor sleep behavior may be an effective mechanism for decreasing substance use. One possible intervention would be a napping program during or after school. Napping has been shown to improve memory [39], [40], [41], [42], [43], alertness [44], and creativity in young adults [45]. Helping adolescents to get more nocturnal sleep may be challenging. Although the present analysis did not examine morning rise time (the information was not available), future studies should investigate whether the majority of adolescents who are vulnerable to the negative consequences of sleep loss for drug use also have the greatest level of delayed sleep phase. Perhaps, for these individuals, a systematic daytime sleep regimen would be advisable, and the results reported here suggest that benefits of such an intervention could spread to influence friends.

Our results also show that adolescents who are most centrally located in the social network are not only more influential but also the most vulnerable to poor health outcomes. This suggests that anti-drug interventions targeting central individuals may be more effective for reducing drug use across the whole network. In the same vein, sleep interventions may benefit from targeting individuals who are more central to the network, and such interventions could spill over and help reduce drug use in many other individuals. Studies of vaccinations have shown a linear effect between success of vaccination and number of connections [46]. Furthermore, costs of interventions can be reduced substantially when more central individuals are treated [47]. Targeted sleep interventions might be similarly effective, helping to immunize a whole population against drug use. People are connected, and so their health behaviors are connected.

## Supporting Information

Text S1 (0.35 MB DOC)
