# Supplementary material for: The Spread of Sleep Loss Influences Drug Use in Adolescent Social Networks
Source: PLoS One. 2010 Mar 19;5(3):e9775. doi: 10.1371/journal.pone.0009775 (PMC2841645; doi:10.1371/journal.pone.0009775)
Supplement: Text S1 — (0.35 MB DOC) [file pone.0009775.s001.doc]

**Supporting Information S1 for**

**The Spread of Sleep Behaviour
Influences Drug Use
in Adolescent Social Networks**

Sara C. Mednick, PhD
University of California, San Diego

Nicholas A. Christakis, MD, PhD
Harvard University

James H. Fowler, PhD
University of California, San Diego

**Measuring network centrality.** *Eigenvector centrality* [48] assumes that the centrality of a given individual is an increasing function of the centralities of all the individuals to whom he or she is connected. While this is an intuitive way to think about which subjects might be better connected, it yields a practical problem—how do we simultaneously estimate the centrality of all subjects in the network? Let *a_ij_* equal 1 if subjects *i* and *j* have a social connection and 0 if they do not. Furthermore, let *x* be a vector of centrality scores so that each subject’s centrality $x_{j}$ is proportional to the sum of the centralities of the subjects to whom they are connected: $\lambda x_{i}=a_{1i}x_{1}+a_{2i}x_{2}+\cdots+a_{ni}x_{n}$. This yields *n* equations, which can be represented as $\lambda x=A^{T}x$. The vector of centralities *x* can now be computed since it is an eigenvector of the eigenvalue *λ*. Although there are *n* nonzero solutions to this set of equations, in symmetric matrices, the eigenvector corresponding to the principal eigenvalue is used because it maximizes the accuracy with which the associated eigenvector can reproduce the original social network [49]. To be sure of reaching a solution, we symmetrized all asymmetric relationships in the observed network (i.e., we assumed all friendship ties were mutual).

**Drawing network maps**. The Kamada-Kawai algorithm used to prepare the images in Figure 1 in the paper generates a matrix of shortest network path distances from each node to all other nodes in the network and repositions nodes so as to reduce the sum of the difference between the plotted distances and the network distances [50].

**Statistical Analysis.** The tables that follow show summary statistics (Table S1) and full specifications for prospective GLM models focused on a single observation of each ego (Tables S2-S5) and longitudinal GEE models that analyze each ego-alter pair as an observation (Tables S6-S9). The specification for the GEE model is ego_behaviour_t+1_ = alter_behaviour_t+1_ + alter_behaviour_t_ + ego_behaviour_t_ + covariates, and the independence error structure controls for multiple observations of the same ego. The sample size, *N*, shown in Tables S6-S9 reflects the total number of all such ego-alter pairings, allowing for the possibility that a given person can have multiple ties. Missing data was imputed using Amelia, a multiple imputation procedure [51] and all model analyses summarize results carried out on 10 multiply-imputed data sets to derive coefficients and standard errors.

We explored the sensitivity of our results to model specification by conducting other analyses (not shown here) each of which had various strengths and limitations, but none of which yielded substantially different results than those presented here. For example, we studied how multiple observations on some principals affected the standard errors of our models. Huber-White sandwich estimates with clustering on the egos yielded very similar standard errors.

**Testing Mediation.**  A variable M mediates the relationship between an independent variable X and a dependent variable Y if (1) X significantly predicts Y, (2) X significantly predicts M, and (3) M significantly predicts Y controlling for X [52]. A formal test of mediation, called the Sobel test, determines whether the indirect effect is significantly different from zero [52,53]. An assumption of the test is that the indirect effect is distributed normally; however, this assumption has been shown to be problematic [29,30,54]. We therefore estimate the indirect effect via a bootstrap procedure in Table 3 of the main text and Table S8 here that does not depend on this assumption [29,30]. The results show that ego sleep significantly mediates the relationship between alter sleep and ego drug use, but alter drug use does not significantly mediate the relationship. This suggests that alter sleep influences ego drug use via the spread of sleep behaviour.

**Table S1. Summary Statistics**

|  | *Wave I* | | | | *Wave II* | | | |
| --- | --- | --- | --- | --- | --- | --- | --- | --- |
|  | *Mean* | *SD* | *Min* | *Max* | *Mean* | *SD* | *Min* | *Max* |
| *Hours of Sleep Per Night, Ego* | 7.78 | 1.35 | 1 | 15 | 7.60 | 1.38 | 1 | 23 |
| *Hours of Sleep Per Night, Alter* | 7.78 | 1.35 | 1 | 15 | 7.61 | 1.38 | 1 | 23 |
| *Sleeps ≤7 Hours Per Night, Ego* | 0.38 | 0.49 | 0 | 1 | 0.44 | 0.50 | 0 | 1 |
| *Sleeps ≤7 Hours Per Night, Alter* | 0.38 | 0.49 | 0 | 1 | 0.44 | 0.50 | 0 | 1 |
| *No. Marijuana Uses in Last 30 Days, Ego* | 1.06 | 4.50 | 0 | 30 | 1.37 | 5.11 | 0 | 30 |
| *No. Marijuana Uses in Last 30 Days, Alter* | 1.07 | 4.55 | 0 | 30 | 1.37 | 5.11 | 0 | 30 |
| *Used Marijuana in Last 30 Days, Ego* | 0.13 | 0.34 | 0 | 1 | 0.15 | 0.36 | 0 | 1 |
| *Used Marijuana in Last 30 Days, Alter* | 0.13 | 0.34 | 0 | 1 | 0.15 | 0.36 | 0 | 1 |
| *Number of Times Nominated as Friend* | 0.71 | 1.49 | 0 | 15 | 0.72 | 1.50 | 0 | 15 |
| *Total Number of Social Contacts* | 2.19 | 2.13 | 1 | 18 | 2.19 | 2.13 | 1 | 18 |
| *Eigenvector Centrality* | 0.03 | 0.09 | 0 | 0.71 | 0.03 | 0.09 | 0 | 0.71 |
| *No. Social Contacts Who Sleep >7 Hours* | 0.89 | 1.50 | 0 | 12 |  |  |  |  |
| *No. Social Contacts Who Sleep ≤7 Hours* | 0.63 | 1.25 | 0 | 12 |  |  |  |  |
| *No. Social Contacts Who Use Marijuana* | 0.24 | 0.75 | 0 | 10 |  |  |  |  |
| *No. Social Contacts Who Do Not Use* | 1.27 | 1.98 | 0 | 16 |  |  |  |  |
| *Age* | 15.81 | 1.59 | 11 | 21 |  |  |  |  |
| *Female* | 0.51 | 0.50 | 0 | 1 |  |  |  |  |
| *Household Income (1000s of Dollars)* | 46.06 | 52.21 | 0 | 999 |  |  |  |  |
| *Parent’s Education* | 5.45 | 2.40 | 0 | 9 |  |  |  |  |
| *Hispanic* | 0.17 | 0.38 | 0 | 1 |  |  |  |  |
| *Black* | 0.23 | 0.42 | 0 | 1 |  |  |  |  |
| *Asian* | 0.07 | 0.26 | 0 | 1 |  |  |  |  |

*Note:* Parent’s education is a 10 item scale (0 = never went to school; 1 = 8th grade or less; 2 = more than 8th grade, but did not graduate from high school; 3 = went to a business, trade, or vocational school instead of high school; 4 = high school graduate; 5 = completed a GED; 6 = went to a business, trade or vocational school after high school; 7 = went to college, but did not graduate; 8 = graduated from a college or university; 9 = professional training beyond a 4-year college or university).

**Table S2. Sleep and Centrality**

|  | ***Dependent Variable:*** | | | | | |
| --- | --- | --- | --- | --- | --- | --- |
|  | *Currently  Sleeps ≤7 Hours* | | | *Current*  *Network Centrality* | | |
|  | *Coef* | *SE* | *p* | *Coef* | *SE* | *p* |
| *Previously Slept ≤7 Hours* | 1.24 | 0.06 | 0.00 | -0.00 | 0.00 | 0.31 |
| *Previous Network Centrality* | 0.67 | 0.31 | 0.03 | -0.02 | 0.02 | 0.27 |
| *Ego Age* | 0.20 | 0.02 | 0.00 | -0.01 | 0.00 | 0.00 |
| *Ego Female* | 0.14 | 0.06 | 0.02 | -0.00 | 0.00 | 0.29 |
| *Ego’s Household Income* | 0.00 | 0.00 | 0.29 | 0.00 | 0.00 | 0.99 |
| *Mother’s Education* | 0.04 | 0.01 | 0.00 | 0.00 | 0.00 | 0.08 |
| *Ego Hispanic* | 0.06 | 0.08 | 0.44 | 0.00 | 0.00 | 0.59 |
| *Ego Black* | 0.17 | 0.07 | 0.02 | -0.00 | 0.00 | 0.31 |
| *Ego Asian* | 0.20 | 0.12 | 0.09 | -0.01 | 0.01 | 0.40 |
| *Constant* | -4.33 | 0.33 | 0.00 | 0.15 | 0.02 | 0.00 |
| *Deviance* | 7044 |  |  | 34 |  |  |
| *Null Deviance* | 7780 |  |  | 35 |  |  |
| *N* | 5663 |  |  | 4053 |  |  |

Results for logistic regression of ego sleep behaviour at Wave II (1 = sleeps ≤7 hours, 0 = sleeps >7 hours) on Wave I covariates is shown in first three columns. In the second and third set of three columns we present results of ordinary least squares regression of network variables at Wave II on covariates in Wave I. The results show that network structure influences sleep behaviour but not vice versa.

**Table S3. Marijuana Use and Centrality**

|  | ***Dependent Variable:*** | | | | | |
| --- | --- | --- | --- | --- | --- | --- |
|  | *Currently  Uses Marijuana* | | | *Current*  *Network Centrality* | | |
|  | Coef | SE | p | Coef | SE | p |
| *Previously Used Marijuana* | 2.29 | 0.09 | 0.00 | 0.00 | 0.00 | 0.57 |
| *Previous Network Centrality* | 0.05 | 0.44 | 0.91 | -0.02 | 0.02 | 0.27 |
| *Ego Age* | 0.05 | 0.03 | 0.09 | -0.01 | 0.00 | 0.00 |
| *Ego Female* | -0.07 | 0.08 | 0.35 | 0.00 | 0.00 | 0.26 |
| *Ego’s Household Income* | -0.00 | 0.00 | 0.42 | 0.00 | 0.00 | 0.98 |
| *Mother’s Education* | 0.04 | 0.02 | 0.04 | 0.00 | 0.00 | 0.10 |
| *Ego Hispanic* | 0.23 | 0.11 | 0.04 | 0.00 | 0.00 | 0.59 |
| *Ego Black* | -0.16 | 0.11 | 0.11 | 0.00 | 0.00 | 0.23 |
| *Ego Asian* | -0.10 | 0.17 | 0.54 | -0.01 | 0.01 | 0.38 |
| *Constant* | -2.92 | 0.46 | 0.00 | 0.16 | 0.02 | 0.00 |
| *Deviance* | 4175 |  |  | 34 |  |  |
| *Null Deviance* | 4859 |  |  | 35 |  |  |
| *N* | 5497 |  |  | 4013 |  |  |

Results for logistic regression of ego drug use behaviour at Wave II (1 = used marijuana in past 30 days, 0 = did not use) on Wave I covariates is shown in first three columns. In the second and third set of three columns we present results of ordinary least squares regression of network variables at Wave II on covariates in Wave I. The results suggest that drug use is associated with more friendship nominations, but the newly attracted friends are peripheral since drug use has no effect on network centrality.

**Table S4. Ego and Alter Sleep in Friends, By Sample and Variable Type**

|  | ***Dependent Variable: Ego’s Current Sleep Behaviour*** | | | | | | | | |
| --- | --- | --- | --- | --- | --- | --- | --- | --- | --- |
|  | *Full Sample,*  *Sleep ≤ 7 Hours* | | | *Single School,*  *Sleep ≤ 7 Hours* | | | *Full Sample,*  *Hours of Sleep* | | |
|  | *Coef* | *SE* | *p* | *Coef* | *SE* | *p* | *Coef* | *SE* | *p* |
| *Alter Current Sleep Behaviour* | 0.19 | 0.06 | 0.00 | 0.30 | 0.15 | 0.04 | 0.07 | 0.01 | 0.00 |
| *Alter Previous Sleep Behaviour* | 0.14 | 0.07 | 0.04 | -0.13 | 0.17 | 0.45 | 0.02 | 0.01 | 0.28 |
| *Ego Previous Sleep Behaviour* | 1.48 | 0.08 | 0.00 | 1.57 | 0.24 | 0.00 | 0.35 | 0.02 | 0.00 |
| *Ego Female* | 0.15 | 0.08 | 0.05 | -0.03 | 0.23 | 0.91 | -0.13 | 0.04 | 0.00 |
| *Ego Age* | 0.20 | 0.03 | 0.00 | 0.19 | 0.12 | 0.10 | -0.11 | 0.01 | 0.00 |
| *Ego’s Household Income* | 0.00 | 0.00 | 0.49 | -0.00 | 0.00 | 0.28 | 0.00 | 0.00 | 0.60 |
| *Mother’s Education* | -0.01 | 0.02 | 0.62 | -0.09 | 0.06 | 0.12 | 0.00 | 0.01 | 0.79 |
| *Ego Hispanic* | -0.22 | 0.12 | 0.06 | -1.43 | 0.80 | 0.07 | 0.04 | 0.06 | 0.55 |
| *Ego Black* | 0.11 | 0.10 | 0.27 | ----- | ----- | ----- | -0.06 | 0.06 | 0.32 |
| *Ego Asian* | 0.22 | 0.14 | 0.13 | 1.19 | 1.18 | 0.31 | -0.18 | 0.07 | 0.01 |
| *Constant* | -4.45 | 0.49 | 0.00 | -3.22 | 2.13 | 0.13 | 6.26 | 0.43 | 0.00 |
| *Deviance* | 1224 |  |  | 207 |  |  | 8498 |  |  |
| *Null Deviance* | 1465 |  |  | 243 |  |  | 10471 |  |  |
| *N* | 5913 |  |  | 974 |  |  | 5913 |  |  |

Results for logistic regression of ego sleep behaviour at Wave II (1 = sleeps >7 hours, 0 = sleeps ≤7 hours) are shown in the first two sets of three columns. Results for ordinary least squares regression of number of hours of sleep at Wave II are shown in the third set of three columns. The model in the second set of three columns is restricted to observations from the largest school in the sample (number 58) in order to show full sample results are not driven by between-school variation (there were no black students in this school). Models were estimated using a general estimating equation with clustering on the ego and an independent working covariance structure. Models with an exchangeable correlation structure yielded poorer fit. Fit statistics show sum of squared deviance between predicted and observed values for the model and a null model with no covariates.

**Table S5. Ego and Alter Drug Use in Friends, By Sample and Variable Type**

|  | ***Dependent Variable: Ego’s Current Marijuana Behaviour*** | | | | | | | | |
| --- | --- | --- | --- | --- | --- | --- | --- | --- | --- |
|  | *Full Sample,*  *Recently Used* | | | *Single School,*  *Recently Used* | | | *Full Sample,  Number of Uses* | | |
|  | *Coef* | *SE* | *p* | *Coef* | *SE* | *p* | *Coef* | *SE* | *p* |
| *Alter Current Marijuana Behaviour* | 0.84 | 0.11 | 0.00 | 0.71 | 0.22 | 0.00 | 0.14 | 0.02 | 0.00 |
| *Alter Previous Marijuana Behaviour* | 0.77 | 0.12 | 0.00 | 0.76 | 0.21 | 0.00 | 0.00 | 0.02 | 0.92 |
| *Ego Previous Marijuana Behaviour* | 2.62 | 0.13 | 0.00 | 2.68 | 0.31 | 0.00 | 0.66 | 0.05 | 0.00 |
| *Ego Female* | -0.17 | 0.11 | 0.14 | 0.05 | 0.31 | 0.86 | -0.39 | 0.12 | 0.00 |
| *Ego Age* | 0.03 | 0.04 | 0.39 | -0.05 | 0.16 | 0.77 | 0.05 | 0.03 | 0.12 |
| *Ego’s Household Income* | 0.00 | 0.00 | 0.01 | -0.02 | 0.01 | 0.00 | 0.00 | 0.00 | 0.00 |
| *Mother’s Education* | 0.03 | 0.03 | 0.19 | 0.00 | 0.07 | 0.99 | 0.02 | 0.03 | 0.56 |
| *Ego Hispanic* | 0.01 | 0.17 | 0.94 | 0.78 | 0.83 | 0.35 | -0.27 | 0.17 | 0.11 |
| *Ego Black* | -0.11 | 0.17 | 0.52 | ----- | ----- | ----- | -0.33 | 0.14 | 0.02 |
| *Ego Asian* | 0.05 | 0.23 | 0.83 | 0.14 | 0.60 | 0.82 | -0.25 | 0.18 | 0.17 |
| *Constant* | -3.09 | 0.70 | 0.00 | -0.99 | 3.22 | 0.76 | 0.37 | 0.63 | 0.56 |
| *Deviance* | 520 |  |  | 112 |  |  | 83338 |  |  |
| *Null Deviance* | 702 |  |  | 174 |  |  | 132298 |  |  |
| *N* | 5913 |  |  | 974 |  |  | 5913 |  |  |

Results for logistic regression of ego drug use behaviour at Wave II (1 = used marijuana in past 30 days, 0 = did not use) are shown in the first two sets of three columns. Results for ordinary least squares regression of number times ego uses marijuana at Wave II are shown in the third set of three columns. The model in the second set of three columns is restricted to observations from the largest school in the sample (number 58) in order to show full sample results are not driven by between-school variation (there were no black students in this school). Models were estimated using a general estimating equation with clustering on the ego and an independent working covariance structure. Models with an exchangeable correlation structure yielded poorer fit. Fit statistics show sum of squared deviance between predicted and observed values for the model and a null model with no covariates.

**Table S6. Ego and Alter Sleep, By Relationship Type**

|  | ***Dependent Variable: Ego Currently Sleeps ≤7 Hours*** | | | | | | | | |
| --- | --- | --- | --- | --- | --- | --- | --- | --- | --- |
|  | *Alter-Perceived Friends* | | | *Mutual*  *Friends* | | | *Siblings* | | |
|  | *Coef* | *SE* | *p* | *Coef* | *SE* | *p* | *Coef* | *SE* | *p* |
| *Alter Currently Sleeps ≤7 Hours* | 0.08 | 0.09 | 0.35 | 0.33 | 0.13 | 0.01 | 0.46 | 0.07 | 0.00 |
| *Alter Previously Slept ≤7 Hours* | -0.03 | 0.09 | 0.78 | 0.13 | 0.14 | 0.35 | 0.08 | 0.07 | 0.21 |
| *Ego Previously Slept ≤7 Hours* | 0.63 | 0.09 | 0.00 | 1.35 | 0.16 | 0.00 | 1.18 | 0.07 | 0.00 |
| *Ego Female* | -0.15 | 0.09 | 0.09 | 0.10 | 0.16 | 0.51 | 0.01 | 0.07 | 0.83 |
| *Ego Age* | 0.22 | 0.03 | 0.00 | 0.26 | 0.06 | 0.00 | 0.15 | 0.02 | 0.00 |
| *Ego’s Household Income* | 0.00 | 0.00 | 0.40 | 0.00 | 0.00 | 0.37 | 0.00 | 0.00 | 0.91 |
| *Mother’s Education* | 0.00 | 0.02 | 0.83 | 0.01 | 0.04 | 0.80 | -0.01 | 0.02 | 0.74 |
| *Ego Hispanic* | -0.03 | 0.14 | 0.85 | -0.32 | 0.23 | 0.16 | -0.06 | 0.10 | 0.52 |
| *Ego Black* | -0.03 | 0.10 | 0.80 | 0.07 | 0.24 | 0.77 | 0.25 | 0.08 | 0.00 |
| *Ego Asian* | 0.04 | 0.13 | 0.78 | 0.08 | 0.29 | 0.78 | 0.04 | 0.13 | 0.74 |
| *Constant* | -3.85 | 1.08 | 0.00 | -5.53 | 1.11 | 0.00 | -3.53 | 0.38 | 0.00 |
| *Deviance* | 681 |  |  | 264 |  |  | 1056 |  |  |
| *Null Deviance* | 727 |  |  | 317 |  |  | 1205 |  |  |
| *N* | 2909 |  |  | 1279 |  |  | 4904 |  |  |

Results for logistic regression of ego sleep behaviour at Wave II (1 = sleeps >7 hours, 0 = sleeps ≤7 hours) on three different samples, alter-perceived friends (first three columns), mutual friends (second three columns), and siblings (last three columns). Models were estimated using a general estimating equation with clustering on the ego and an independent working covariance structure. Models with an exchangeable correlation structure yielded poorer fit. Fit statistics show sum of squared deviance between predicted and observed values for the model and a null model with no covariates.

**Table S7. Ego and Alter Drug Use, By Relationship Type**

|  | ***Dependent Variable: Ego Currently Uses Marijuana*** | | | | | | | | |
| --- | --- | --- | --- | --- | --- | --- | --- | --- | --- |
|  | *Alter-Perceived Friends* | | | *Mutual*  *Friends* | | | *Siblings* | | |
|  | *Coef* | *SE* | *p* | *Coef* | *SE* | *p* | *Coef* | *SE* | *p* |
| *Alter Currently Uses Marijuana* | 0.51 | 0.22 | 0.02 | 1.37 | 0.23 | 0.00 | 0.64 | 0.12 | 0.00 |
| *Alter Previously Used Marijuana* | -0.44 | 0.20 | 0.03 | 0.39 | 0.26 | 0.14 | 0.51 | 0.13 | 0.00 |
| *Ego Previously Used Marijuana* | 1.14 | 0.12 | 0.00 | 2.03 | 0.28 | 0.00 | 1.94 | 0.11 | 0.00 |
| *Ego Female* | -0.08 | 0.11 | 0.46 | -0.24 | 0.23 | 0.29 | -0.18 | 0.09 | 0.05 |
| *Ego Age* | 0.07 | 0.04 | 0.05 | 0.10 | 0.09 | 0.27 | 0.00 | 0.03 | 1.00 |
| *Ego’s Household Income* | 0.00 | 0.00 | 0.51 | -0.01 | 0.00 | 0.04 | 0.00 | 0.00 | 0.51 |
| *Mother’s Education* | 0.01 | 0.02 | 0.68 | 0.02 | 0.06 | 0.73 | 0.02 | 0.02 | 0.25 |
| *Ego Hispanic* | 0.08 | 0.13 | 0.55 | 0.02 | 0.37 | 0.96 | 0.26 | 0.13 | 0.05 |
| *Ego Black* | -0.01 | 0.13 | 0.96 | -0.12 | 0.48 | 0.80 | -0.07 | 0.11 | 0.57 |
| *Ego Asian* | -0.32 | 0.15 | 0.03 | -0.05 | 0.36 | 0.89 | -0.22 | 0.21 | 0.29 |
| *Constant* | -2.69 | 1.32 | 0.04 | -3.76 | 1.69 | 0.03 | -2.12 | 0.52 | 0.00 |
| *Deviance* | 470 |  |  | 123 |  |  | 568 |  |  |
| *Null Deviance* | 502 |  |  | 159 |  |  | 675 |  |  |
| *N* | 2909 |  |  | 1279 |  |  | 4904 |  |  |

Results for logistic regression of ego drug use behaviour at Wave II (1 = used marijuana in past 30 days, 0 = did not use) on three different samples, alter-perceived friends (first three columns), mutual friends (second three columns), and siblings (last three columns). Models were estimated using a general estimating equation with clustering on the ego and an independent working covariance structure. Models with an exchangeable correlation structure yielded poorer fit. Fit statistics show sum of squared deviance between predicted and observed values for the model and a null model with no covariates.

**Table S8. Alter Drug Use Does Not Mediate Relationship Between Alter Sleep and Ego Drug Use**

|  | ***Dependent Variable:*** | | | | | | | | |
| --- | --- | --- | --- | --- | --- | --- | --- | --- | --- |
|  | *Ego Currently Uses Marijuana* | | | *Alter Currently Uses Marijuana* | | | *Ego Currently Uses Marijuana* | | |
|  | Coef | SE | p | Coef | SE | p | Coef | SE | p |
| *Alter Currently Sleeps ≤7 Hours* | 0.20 | 0.10 | 0.04 | 0.15 | 0.07 | 0.04 | 0.18 | 0.10 | 0.07 |
| *Alter Currently Uses Marijuana* | ----- | ----- | ----- | ----- | ----- | ----- | 0.83 | 0.11 | 0.00 |
| *Alter Previously Used Marijuana* | 0.99 | 0.11 | 0.00 | 1.39 | 0.09 | 0.00 | 0.77 | 0.12 | 0.00 |
| *Ego Previously Used Marijuana* | 2.65 | 0.13 | 0.00 | 0.59 | 0.11 | 0.00 | 2.62 | 0.13 | 0.00 |
| *Ego Female* | -0.17 | 0.11 | 0.14 | -0.01 | 0.07 | 0.90 | -0.17 | 0.11 | 0.13 |
| *Ego Age* | 0.03 | 0.04 | 0.47 | 0.06 | 0.03 | 0.02 | 0.02 | 0.04 | 0.61 |
| *Ego’s Household Income* | -0.00 | 0.00 | 0.01 | 0.00 | 0.00 | 0.05 | -0.00 | 0.00 | 0.01 |
| *Mother’s Education* | 0.03 | 0.03 | 0.22 | -0.00 | 0.02 | 0.86 | 0.03 | 0.03 | 0.21 |
| *Ego Hispanic* | -0.02 | 0.17 | 0.93 | -0.22 | 0.12 | 0.06 | 0.02 | 0.17 | 0.91 |
| *Ego Black* | -0.10 | 0.17 | 0.54 | 0.09 | 0.11 | 0.41 | -0.11 | 0.17 | 0.51 |
| *Ego Asian* | 0.04 | 0.22 | 0.86 | -0.08 | 0.15 | 0.59 | 0.05 | 0.23 | 0.84 |
| *Constant* | -2.97 | 0.70 | 0.00 | -2.99 | 0.71 | 0.00 | -2.96 | 0.71 | 0.00 |
| *Deviance* | 528 |  |  | 837 |  |  | 519 |  |  |
| *Null Deviance* | 702 |  |  | 922 |  |  | 702 |  |  |
| *N* | 5913 |  |  | 5913 |  |  | 5913 |  |  |

First three columns show logistic regression model of ego’s marijuana use behaviour (the outcome variable) on alter’s sleep behaviour (the explanatory variable). Second three columns show logistic regression model of alter’s drug behaviour (the mediator variable) on alter’s sleep behaviour (the explanatory variable). Last three columns show logistic regression model of ego’s marijuana use behaviour (the outcome variable) on alter’s drug behaviour (the mediator variable) controlling for alter’s sleep behaviour (the explanatory variable). Models were estimated using a general estimating equation with clustering on the ego and an independent working covariance structure. Models with an exchangeable correlation structure yielded poorer fit. Fit statistics show sum of squared deviance between predicted and observed values for the model and a null model with no covariates. A bootstrap procedure that takes into account uncertainty of both the effect of the independent variable on the mediator and the mediator on the outcome variable shows that alter drug use is not a significant mediator (*p*=0.08)

**Table S9. Alter Drug Use Does Not Predict Ego Sleep**

|  | ***Dependent Variable:***  ***Ego Currently Sleeps Enough*** | | | | | |
| --- | --- | --- | --- | --- | --- | --- |
|  | *Marijuana*  *Contagion Model* | | | *Sleep*  *Contagion Model* | | |
|  | *Coef* | *SE* | *p* | *Coef* | *SE* | *p* |
| *Alter Currently Uses Marijuana* | 0.06 | 0.08 | 0.42 | 0.08 | 0.08 | 0.36 |
| *Alter Previously Used Marijuana* | 0.02 | 0.08 | 0.83 | ----- | ----- | ----- |
| *Ego Previously Used Marijuana* | 0.44 | 0.12 | 0.00 | ----- | ----- | ----- |
| *Alter Previously Slept ≤7 Hours* | ----- | ----- | ----- | 0.16 | 0.06 | 0.01 |
| *Ego Previously Slept ≤7 Hours* | ----- | ----- | ----- | 1.47 | 0.08 | 0.00 |
| *Ego Female* | 0.24 | 0.07 | 0.00 | 0.15 | 0.08 | 0.04 |
| *Ego Age* | 0.29 | 0.02 | 0.00 | 0.21 | 0.03 | 0.00 |
| *Ego’s Household Income* | 0.00 | 0.00 | 0.40 | 0.00 | 0.00 | 0.60 |
| *Mother’s Education* | -0.00 | 0.02 | 0.76 | -0.01 | 0.02 | 0.62 |
| *Ego Hispanic* | -0.12 | 0.11 | 0.26 | -0.22 | 0.12 | 0.05 |
| *Ego Black* | 0.34 | 0.10 | 0.00 | 0.11 | 0.10 | 0.25 |
| *Ego Asian* | 0.52 | 0.14 | 0.00 | 0.23 | 0.14 | 0.11 |
| *Constant* | -5.57 | 0.46 | 0.00 | -4.55 | 0.48 | 0.00 |
| *Deviance* | 1374 |  |  | 1226 |  |  |
| *Null Deviance* | 1465 |  |  | 1465 |  |  |
| *N* | 5913 |  |  | 5913 |  |  |

Logistic regression models of alter’s sleep behaviour (the outcome variable) on ego’s marijuana use behaviour (the outcome variable). The first three columns include controls for alter marijuana use as a mediator. The second three columns include controls for ego sleep behaviour as a mediator. Models were estimated using a general estimating equation with clustering on the ego and an independent working covariance structure. Models with an exchangeable correlation structure yielded poorer fit. Fit statistics show sum of squared deviance between predicted and observed values for the model and a null model with no covariates. The results show there is no direct effect of alter drug use on ego sleep behaviour.
